# Evaluation of sit-stand workstations in an office setting: a randomised controlled trial

**DOI:** 10.1186/s12889-015-2469-8

**Published:** 2015-11-19

**Authors:** Lee E. F. Graves, Rebecca C. Murphy, Sam O. Shepherd, Josephine Cabot, Nicola D. Hopkins

**Affiliations:** Research Institute for Sport and Exercise Science, Liverpool John Moores University, Liverpool, UK; Research Institute for Sport and Exercise Science, Physical Activity Exchange, Liverpool John Moores University, 62 Great Crosshall Street, Liverpool, L3 2AT UK

**Keywords:** Sitting, Intervention, Desks, Workplace, Cardiovascular, Metabolic, Qualitative

## Abstract

**Background:**

Excessive sitting time is a risk factor for cardiovascular disease mortality and morbidity independent of physical activity. This aim of this study was to evaluate the impact of a sit-stand workstation on sitting time, and vascular, metabolic and musculoskeletal outcomes in office workers, and to investigate workstation acceptability and feasibility.

**Methods:**

A two-arm, parallel-group, individually randomised controlled trial was conducted in one organisation. Participants were asymptomatic full-time office workers aged ≥18 years. Each participant in the intervention arm had a sit-stand workstation installed on their workplace desk for 8 weeks. Participants in the control arm received no intervention. The primary outcome was workplace sitting time, assessed at 0, 4 and 8 weeks by an ecological momentary assessment diary. Secondary behavioural, cardiometabolic and musculoskeletal outcomes were assessed. Acceptability and feasibility were assessed via questionnaire and interview. ANCOVA and magnitude-based inferences examined intervention effects relative to controls at 4 and 8 weeks. Participants and researchers were not blind to group allocation.

**Results:**

Forty-seven participants were randomised (intervention *n* = 26; control *n =* 21). Relative to the control group at 8 weeks, the intervention group had a beneficial decrease in sitting time (−80.2 min/8-h workday (95 % CI = −129.0, −31.4); *p* = 0.002), increase in standing time (72.9 min/8-h workday (21.2, 124.6); *p* = 0.007) and decrease in total cholesterol (−0.40 mmol/L  (−0.79, −0.003); *p* = 0.049). No harmful changes in musculoskeletal discomfort/pain were observed relative to controls, and beneficial changes in flow-mediated dilation and diastolic blood pressure were observed. Most participants self-reported that the workstation was easy to use and their work-related productivity did not decrease when using the device. Factors that negatively influenced workstation use were workstation design, the social environment, work tasks and habits.

**Conclusion:**

Short-term use of a feasible sit-stand workstation reduced daily sitting time and led to beneficial improvements in cardiometabolic risk parameters in asymptomatic office workers. These findings imply that if the observed use of the sit-stand workstations continued over a longer duration, sit-stand workstations may have important ramifications for the prevention and reduction of cardiometabolic risk in a large proportion of the working population.

**Trial registration:**

ClinicalTrials.gov NCT02496507.

## Background

Office-based workers can spend as much as 10–11 h of a working day in a seated static posture [1], which represents an ergonomic hazard in the physical work environment [2]. Prolonged sitting and total sitting time are associated with poor metabolic health [3], greater risk of chronic diseases [4] and premature mortality [5]. Office workers are therefore at increased risk of the negative health outcomes associated with excessive sitting including obesity, insulin resistance, cardiovascular disease, depression and chronic back and neck pain [6]. The preventative role of moderate-to-vigorous physical activity in the development of these conditions is irrefutable [7]. Critically however, the association between sitting time and poor health remains after accounting for moderate-to-vigorous physical activity [6]. This presents a compelling rationale for public health programmes that target sitting reduction in the workplace, as supported by a recent expert statement [8]. Such programmes may also help organisations reduce costs associated with healthcare and absenteeism [8].

The metabolic impact of breaking up prolonged sitting with standing or light activity has been investigated in acute, lab-based studies. Findings indicate favourable changes in postprandial glucose and insulin excursions [9, 10], suggesting there is potential for long-term sit reduction interventions to significantly impact metabolic disease risk. Two randomised controlled trials (RCTs) have attempted to elucidate the impact of reduced workplace sitting on surrogate health markers in workers [11, 12]. Reduced sitting time caused positive changes in high-density lipoprotein (HDL) cholesterol concentrations following 3 months of using a sit-stand workstation [11], however no changes in fasting blood glucose, triglyceride and total cholesterol concentrations were reported [11, 12]. This paucity of literature suggests more research is warranted to better understand the metabolic effects of reduced workplace sitting.

Although epidemiological evidence clearly highlights the link between sitting time and cardiovascular morbidity and mortality [5, 13, 14], the cardiovascular effects of reducing sitting are yet to be assessed or confirmed experimentally. Endothelial dysfunction, an early sign of atherosclerotic disease, is considered a manifestation of the compound impact of traditional risk factors. Importantly, endothelial dysfunction strongly and independently predicts cardiovascular events [15, 16] and is reversed by interventions known to diminish cardiovascular risk [17–19]. Assessment of endothelial function allows presence and magnitude of cardiovascular risk to be quantified prior to the development of overt disease and in response to interventions targeting sitting time.

The sustainability of sit-stand workstations will be determined by many factors, including feasibility and the impact on work performance and practices. Some, albeit limited evidence demonstrates inter-participant variation in the way that sit-stand workstations are utilised in offices [20, 21] and knowledge concerning feasibility and acceptability of sit-to-stand workstations is suggestive of inter-participant variation in determinants of initiation, maintenance and discontinued use [20–22]. To date, no trial has qualitatively assessed the acceptability and feasibility of sit-stand workstations and associated quantitative changes in behaviour, and markers of vascular and metabolic disease risk in office workers. This data would significantly add to the literature by providing a more comprehensive understanding of the potential for sit-stand workstations to be accepted and used in large-scale workplace health programmes.

The aim of this 8-week intervention was to evaluate changes in workplace sitting following installation of a sit-stand workstation, compared to normal working conditions. Associated effects on vascular and metabolic disease risk markers were evaluated, as was the acceptability and feasibility of sit-stand workstations in a real office setting.

## Method

### Study design

Data for this two-arm parallel-group RCT was collected between October-December 2013. Treatment arms included a sit-stand workstation intervention group (each participant received a sit-stand workstation) and a control group (usual practice). Liverpool John Moores University ethically approved the trial. Participants and researchers were not blinded to group allocation.

### Recruitment

#### Organisation level

**Fig. 1:**
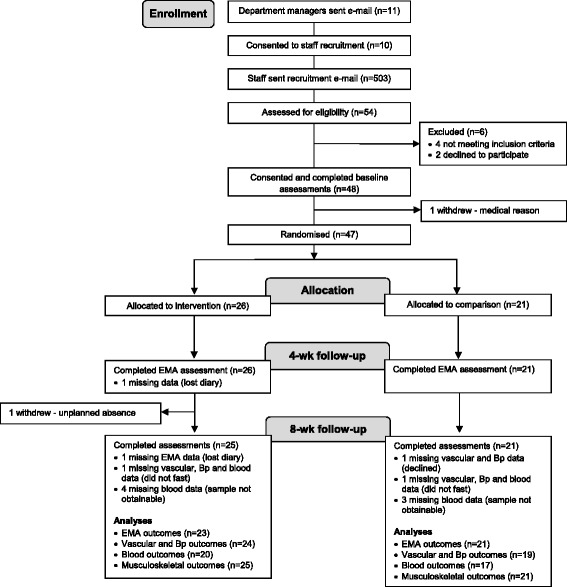
Consort flow diagram of enrolment, allocation, follow-up and analyses

Office workers from one organisation (Liverpool John Moores University, Liverpool, UK) were approached by the research team in August-September 2013. Consent was sought from 11 departmental managers for employee recruitment, installation of sit-stand workstations, study contact and laboratory visits during work time (Fig. 1). Departments were located across four buildings with varying office layout (open-plan, individual offices or a combination). Employees within the approached departments were predominantly administrative staff.

#### Individual level

Via an email from the research team, all employees in consenting departments received an overview of the study and participant information sheet, and were invited to a study information session (two sessions were organised per department). Employees were given 2 weeks to express interest. Interested employees were screened for the following eligibility criteria by the research team via telephone: a) full-time member of staff, b) access to a work telephone and desktop computer with internet, c) no cardiovascular or metabolic disease, d) not taking any medication, e) not pregnant and, f) no planned absence >1 week during the trial. If inclusion criteria were met, written informed consent was obtained and baseline assessments scheduled. There was no racial or gender bias in the selection of participants.

### Group assignment and intervention

Following baseline assessments, participants were assigned by one member of the research team to a treatment arm using a randomised block design [23] and random number table. Departments served as blocks and participants within departments were randomly assigned at the individual-level to an arm [23]. Assignment of individual participants within each department alternated between arms (i.e. intervention, control, intervention, control…).

#### Intervention group

After baseline assessments, each participant had a sit-stand workstation installed on their existing workplace desk. A single (manufacturer’s suggested retail price £360) or dual (£375) monitor WorkFit-A with Worksurface + workstation was installed, dependent on the number of monitors the participant had. The computer monitor(s) and keyboard were housed on the workstation and the workstation could be quickly raised up and down by hand to enable seated or standing work. Participants were not prescribed an amount of time to use the station. Ergotron Ltd (www.ergotron.com) provided and installed the workstations in the standing position and gave participants basic face-to-face training and ergonomic information on correct workstation use. Participants received a web link to manufacturer ergonomic guidelines via an email from the research team (http://www.ergotron.com/tabid/305/language/en-AU/Default.aspx). No other behaviour change techniques were delivered, other than provision of the workstation. After end-intervention data collection, manufacturer staff uninstalled the workstations.

#### Control group

Participants were asked to maintain their normal work practices and received no intervention. Participants were offered the opportunity to have a sit-stand workstation installed for 8 weeks after all data collection.

### Data collection

At baseline, 4 weeks (mid-intervention) and 8 weeks (end-intervention), participants’ office-based behaviours were assessed via ecological momentary assessment (EMA) diaries. At baseline and 8 weeks, participants attended University laboratories in the morning for individual assessments. Prior to laboratory visits, participants were required to fast for a minimum of 8 h, avoid the consumption of alcohol for 12 h, and avoid strenuous exercise for 24 h.

### Outcome measures

#### Sitting, standing and walking time

The EMA diary assessed time spent sitting (primary outcome), standing, walking and in other activities during work hours over 5 days (Monday-Friday). At 15-minute intervals participants used a paper-based diary to record their main behaviour in response to the question: “What are you doing right now?” The behaviour options were sitting, standing, walking or other. If other was selected, participants were instructed to write the activity they were doing. EMA has been reported successfully in recent behavioural research [24, 25], including workplace research [26]. To promote compliance and minimise data loss, participants used the EMA diary to record the time they started and finished work each day, and consenting participants received one prompt to complete the diary via text and/or email at the start of each day from the research team.

Time spent in each behaviour per day (minutes/day: sitting, standing, walking, other) was estimated by multiplying the frequency of recordings by 15, based on the assumption that each behaviour episode occurred for the entire 15 min [25]. This method is assumed to provide valid estimates of time spent in behaviour categories when summed across a day, due to underestimation and overestimation errors cancelling each other out [27]. A diary day was considered valid if data entries were provided for ≥75 % of time spent at work (228/235 days at baseline; 218/235 days at 4 weeks; 193/230 days at 8 weeks). Time spent in each behaviour was calculated for each valid day and means were calculated from valid days. In accordance with previous trials [11, 12, 22] outcomes were standardised to an 8-hour work day to control for variations in work schedules [standardised minutes = outcome minutes * 480/observed workplace minutes]. To be retained for analyses, participants had to provide ≥2 valid days at each time point (met by 23 intervention and 21 control participants).

#### Vascular outcomes

Endothelial dysfunction is an early and integral manifestation of atherosclerotic disease [28] that strongly and independently predicts cardiovascular events in asymptomatic adults [15], and can be measured in the peripheral conduit arteries non-invasively using flow-mediated dilation (FMD). To this end, B-mode images of the brachial artery were obtained in longitudinal section at a reproducible point in the distal third of the upper arm using high resolution ultrasonography (Terason, t3000, Teratech) and a 10–12-MHz probe. Ultrasonic parameters were set to optimize the luminal-arterial wall interface with the focal zone set to the near wall. Once set, these parameters remained constant throughout the session and the probe was held in a constant position. Endothelium-dependent vasodilation was assessed by measuring FMD in response to 5 min of forearm ischaemia [29]. Briefly, a 1-min baseline measurement was taken, then a pneumatic rapid cuff inflator (Hokanson, Bellevue, U.S.A.), placed around the forearm distal to the humeral epicondyle, was inflated to 220 mmHg for 5 min [30]. Recording of the image ceased on inflation of the cuff and recommenced 30 s prior to deflation. Recording continued for a period of 3 min post cuff deflation [30]. Data was analysed post hoc by custom designed automated edge-detection and wall-tracking software, the validity and reproducibility of which have been previously demonstrated [31].

Carotid artery intima media thickness (cIMT) is an early subclinical marker of structural atherosclerosis. Increased cIMT independently predicts cardiovascular events, is correlated with cardiovascular risk factors [32] and as such cIMT is commonly used as a surrogate marker of cardiovascular disease risk. cIMT was measured in the carotid artery using high resolution ultrasound from three different angles over six consecutive cardiac cycles. Subjects were instructed to turn the head laterally, by approximately 90° to the left, and the same investigator undertook all measurements to ensure head positioning and stability were maintained. The common carotid artery was measured 2 cm proximal to the bulbous. A two-dimensional image of the artery was obtained with the near and far wall of the artery displayed as two bright white lines separated by a hypoechogenic space. Post-test analysis was performed using custom designed automated edge-detection and wall-tracking software. A region of interest of at least 1 cm was taken on the first frame of every individual study, including both vessel walls. Detection of the near and far wall lumen edges and the far wall media-adventitia interface was performed on all subsequent frames. The distance from the leading edge of the first bright line of the far wall (lumen-intima interface) to the leading edge of the second bright line (media-adventitia interface) indicated the cIMT [33].

#### Blood sampling

Fasting blood samples were obtained from the antecubital vein of one arm via standard venepuncture technique (Vacutainers Systems, Becton-Dickinson). Samples were collected into vacutainers containing EDTA or lithium heparin and stored on ice until centrifugation for 15 min at 1500 g at 4 °C. Plasma aliquots were stored at −80 °C for subsequent analysis. Plasma glucose, triglycerides and total cholesterol concentrations were determined spectrophotometrically using commercially available kits (Randox Laboratories, Antrim, UK). Each sample was analysed in duplicate.

#### Musculoskeletal outcomes

Using a questionnaire adapted from a previous trial [26], participants rated their current level of discomfort or pain at three sites (lower back, upper back, neck and shoulders) on a Likert scale from 0 (no discomfort) to 10 (extremely uncomfortable).

#### Anthropometric, sociodemographic, work-related and office environment characteristics

Using standard techniques [34] stature was measured to the nearest 0.1 cm using a Leicester Height Measure and body mass to the nearest 0.1 kg using a calibrated mechanical flat scale (both Seca Ltd, Birmingham, UK). Participants wore light clothing and no shoes. Body mass index (BMI) was calculated as mass divided by stature (kg/m^2^). Participants self-reported sociodemographic, work-related and office environment characteristics at baseline (age, gender, ethnicity, marital status, education attainment, smoking history, employment history, job category, office layout, number of people in office).

#### Acceptability and feasibility

At 8 weeks, participants in the intervention arm completed a 19-item five-point Likert scale (1 strongly disagree, 2 disagree, 3 neutral, 4 agree, 5 strongly disagree) adapted from a previous trial [35] to assess the acceptability and feasibility of the sit-stand workstation. Purposive sampling was employed to invite participants of the intervention arm (*n* = 23) to attend a semi-structured interview to discuss their experiences and perspectives of using the workstation. Recruitment emails were sent to all participants in the intervention arm. Seven female participants responded and took part, with interviews facilitated by the second author. The seven participants interviewed did not significantly differ to other intervention arm participants for any baseline characteristic (*p* > 0.05). Focus groups were conducted using a semi-structured interview guide to ensure consistency in interview approach. The semi-structured interview guides were designed to allow freedom in response whilst also ensuring a degree of commonality across the transcripts [36]. Interview questions were developed based upon a review of established literature [37] and the identified aims of the study. Sample questions included; *“Please provide a brief overview of your experience of using the sit-stand workstation?”* (prompts included the use of the workstation during the working day in terms of choice of tasks to stand and complete, patterns of use, time of day selected to stand) and *“Can you reflect on the influence of the workstation on your working practices?”*. Interviews took place in a familiar work setting, during work hours and within a space where participants could be overlooked but not overheard. Interviews lasted on average 13 min (range 8–17 min), were audio recorded and later transcribed verbatim. Verbatim transcripts were read and re-read to allow familiarisation of the data. Content analysis techniques were used to identify core and common themes in the data [38]. The process involved reading and re-reading text and assigning broad thematic codes. The lead (LG) and second author (RM) discussed and debated emerging themes in the data with reference to acceptability and feasibility of workstation use. Therefore a combination of inductive and deductive techniques was used to generate codes. Key emergent themes and participant quotes have been used to ensure authenticity in the represented data.

### Sample size

Allowing for small drop out, the study aimed to recruit 25 participants per arm, and retain 23 participants per arm. A sample size of 23 per arm was chosen a priori to achieve 90 % power (alpha 5 %; two-tailed) to detect a minimum difference of 60 min/8-h workday between arms for workplace sitting time (primary outcome: expected SD of 60 min/day). 60 min was selected based on a recent protocol paper [39] and differences observed in similar trials [22, 26]. Data collection for vascular and metabolic outcomes would provide effect size estimates for power calculations in subsequent trials.

### Statistical analyses

Data was analysed using SPSS version 22 (IBM, New York, USA) with the alpha level set at *p* ≤0.05. Intervention effects were compared at 4 weeks (sitting, standing and walking) and 8 weeks (all outcomes) from baseline using analysis of covariance (ANCOVA). The variable change score (4 or 8 weeks minus baseline) was the dependent variable, with intervention arm (control vs intervention) the independent variable. In all analyses, covariates were the baseline value for the variable to control for any imbalances at baseline [40]. Anthropometric, sociodemographic, work-related and office-environment characteristics were tested as potential confounders for all outcomes. Confounders were entered as covariates if significant associations (*p* ≤0.05) were observed with changes in an outcome and the effect on the mean difference between groups exceeded 20 % [41]. For changes in sitting, standing and walking time, baseline values of the other two behaviours were tested as potential confounders, though no effects on the mean difference between groups exceeded 20 % [41]. Adjusted change scores and 95 % confidence intervals (CIs) for the difference in change between groups are presented unless stated otherwise. Acceptability and feasibility data are reported as medians and quartiles.

#### Missing data and intention-to-treat analysis

Due to participant withdrawal, lost EMA diaries or the inability to conduct assessments, data were missing for all outcomes (Fig. 1). Accordingly, a per-protocol analysis was conducted and participants were excluded from analyses for outcomes they were missing data for. For workplace sitting, standing and walking, the per-protocol analysis was compared with an intention-to-treat analysis, as a sensitivity analysis. To treat missing data, the fully conditional imputation technique and ten imputation sets were used due to a low rate of missing data [42]. Imputation was based on all 47 randomized participants.

#### Minimum important differences analysis

Inferential statistics were ran using minimum clinically important difference principles, described elsewhere [43, 44]. Briefly, this approach makes inferences based on meaningful magnitudes and is recommended alongside hypothesis testing [43, 44]. A spreadsheet (see http://newstats.org/generalize.html) computed the quantitative and qualitative probability that the true effects were beneficial, trivial or harmful, after the outcome statistic, its *p* value, and the smallest/minimal important difference was entered. Minimum important differences for sitting and standing were 60 min/day, and for walking 10 min/day, as guided by a recent protocol paper [39] and differences in similar trials [22, 26]. Minimum important differences for other outcomes were determined through a distribution-based method as a Cohen’s d (standardized difference between change scores between groups) of 0.2 between-subjects standard deviations (SDs) [45]. The SD of pooled baseline data was used to negate the possibility of individual differences from the intervention influencing the SD at 8 weeks. For each effect at 8 weeks, quantitative probabilities for benefit, trivial and harm, and qualitative descriptors are reported. Effects were interpreted as unclear if probabilities for benefit and harm were >5 % [46].

## Results

Forty-seven participants were randomised (Fig. 1). The number of participants analysed for change in workplace sitting time (primary outcome) at 4 and 8 weeks was 44 (23 intervention, 21 control). Participants were predominantly White British women educated at the tertiary level. Ten of the 47 participants were in health-related posts, however potential bias to intervention responsiveness would have been minimised through the randomisation method that evenly allocated these participants to the treatment arms. Groups were comparable at baseline except for a higher proportion of women in the intervention arm (Tables 1, 2 and 3). For all participants combined, sitting, standing and walking time occupied 80 ± 10 %, 9 ± 6 % and 11 ± 7 % of time spent at the workplace, respectively.

**Table 1:** Baseline characteristics by group, presented as mean ± SD or % (n) of group

	Intervention (*n* = 26)	Control (*n* = 21)	All (*n* = 47)
Age (years)	38.8 ± 9.8	38.4 ± 9.3	38.6 ± 9.5
Female	89 (23)	67 (14)	79 (37)
White British	92 (24)	100 (21)	96 (45)
Married	58 (15)	48 (10)	53 (25)
Time at current workplace			
<1 year	0 (0)	0 (0)	0 (0)
1–3 years	8 (2)	19 (4)	13 (6)
>3 years	92 (24)	81 (17)	87 (41)
Tertiary education	100 (26)	91 (19)	96 (45)
Job category			
Manager/Director	31 (8)	14 (3)	23 (11)
Clerical/Services/Other	69 (18)	86 (18)	77 (36)
Number of other people in the office			
0	19 (5)	19 (4)	19 (9)
1–3	12 (3)	0 (0)	6 (3)
>3	69 (18)	81 (17)	75 (35)
Never smoked	77 (20)	76 (16)	77 (36)
Body mass (kg)	67.4 ± 13.8	70.5 ± 16.4	68.8 ± 14.9
Body mass index (kg/m^2^)	24.9 ± 4.4	24.7 ± 4.6	24.8 ± 4.4

**Table 2:** Behavioural outcomes with adjusted between-group differences and quantitative and qualitative inferences^a^

	Intervention (*n* = 23)			Control (*n* = 21)			Adjusted change 0 to 4 week (95 % CI)^b^	Adjusted change 0 to 8 week (95 % CI)^b^	Probability (%) the true effect is beneficial / trivial / harmful	Qualitative inference (8 week)
(minutes/8-hour workday)	Baseline	4 week	8 week	Baseline	4 week	8 week				
Sitting time	385.9 (57.6)	299.2 (93.4)	322.0 (99.3)	387.0 (41.0)	387.5 (78.0)	402.2 (47.9)	−87.6 (−136.8 to −38.3)*	−80.2 (−129.0 to −31.4) *	79/21/0	Benefit likely
Standing time	41.1 (35.0)	141.1 (98.0)	115.4 (111.6)	42.5 (26.0)	61.0 (76.2)	43.7 (50.2)	82.2 (36.5 to 127.8)*	72.9 (21.2 to 124.6)*	69/31/0	Benefit possible
Walking time	53.0 (41.2)	39.7 (33.4)	42.6 (42.3)	50.5 (24.2)	31.4 (24.4)	34.0 (29.1)	7.3 (−8.7 to 23.2)	7.1 (−12.1 to 26.3)	38/58/4	Possibly trivial

^a^Baseline, 4- and 8-week values are unadjusted mean (SD)

^b^Change scores and 95 % CIs are the differences between groups (relative to control) after adjustment by ANCOVA for the baseline value

*Significant (*p* <0.05)

**Table 3:** Cardiometabolic and musculoskeletal outcomes with adjusted between-group differences and quantitative and qualitative inferences^a^

	Intervention		Control		Adjusted change 0 to 8 week (95 % CI)^b^	Probability (%) the true effect is beneficial / trivial / harmful	Qualitative inference
	Baseline	8 week	Baseline	8 week			
Vascular (*n* = 24 I, 19 C)							
FMD (%)	5.98 (2.32)	7.13 (2.42)	5.88 (2.29)	6.13 (2.64)	0.97 (−0.55 to 2.50)	75/22/3	Benefit likely
cIMT (mm)	0.62 (0.07)	0.61 (0.07)	0.58 (0.08)	0.57 (0.08)	0.00 (−0.03 to 0.02)	13/84/3	Likely trivial
Systolic BP (mmHg)	119.1 (13.8)	117.1 (12.5)	117.9 (12.1)	117.3 (9.0)	−1.6 (−7.0 to 3.7)	22/71/7	Unclear
Diastolic BP (mmHg)	73.5 (7.6)	68.9 (8.5)	71.8 (10.7)	70.5 (9.5)	−2.5 (−7.2 to 2.2)	62/35/3	Benefit possible
Blood (*n* = 20 I, 17 C)							
Glucose (mmol/L)	5.30 (0.79)	4.59 (0.84)	4.85 (0.62)	4.49 (0.55)	−0.09 (−0.56 to 0.39)	37/49/14	Unclear
Triglycerides (mmol/L)	1.65 (0.70)	1.61 (0.74)	1.61 (0.64)	1.65 (0.73)	0.11 (−0.23 to 0.45)	6/55/39	Unclear
Cholesterol (mmol/L)	4.45 (0.98)	3.79 (1.05)	3.94 (0.86)	3.78 (0.74)	−0.40 (−0.79 to −0.003)*	82/18/0	Benefit likely
Musculoskeletal discomfort/pain^c^ (*n* = 25 I, 21 C)							
Lower back	2.5 (2.2)	1.8 (2.0)	2.0 (2.0)	1.7 (1.8)	−0.2 (−1.0 to 0.7)	35/50/15	Unclear
Upper back	1.9 (2.3)	1.1 (1.7)	1.2 (1.5)	1.6 (2.3)	−0.9 (−1.9 to 0.2)	83/16/1	Benefit likely
Neck and shoulder	2.6 (2.5)	1.9 (2.4)	2.1 (2.0)	2.2 (2.4)	−0.6 (−1.5 to 0.2)	63/36/1	Benefit possible

*I* intervention group, *C* control group, *FMD* flow-mediated dilation, *cIMT* carotid intima-media thickness, *BP* blood pressure

^a^Baseline and 8-weeks values are unadjusted mean (SD)

^b^Change scores and 95 % CIs are the differences between groups (relative to control) after adjustment by ANCOVA for the baseline value. Triglycerides ANCOVA additionally adjusted for marital status, time at current workplace and job category

^**c**^Values denote the severity of discomfort or pain from 0 (No discomfort) to 10 (Extremely uncomfortable)

*Significant (*p* = 0.049)

### Intervention effects

#### Sitting, standing and walking time

Findings were similar for the per-protocol (Table 2) and intention-to-treat analyses. At 4 and 8 weeks there were clear beneficial reductions in sitting time (intention-to-treat: mean *p* = 0.001 and *p* = 0.002, respectively) and increases in standing time (intention-to-treat: mean *p* = 0.001 and *p* = 0.013, respectively) in the intervention group relative to controls. No between group differences were found for walking at 4 or 8 weeks (intention-to-treat: mean *p* = 0.290 and *p* = 0.408, respectively).

#### Vascular outcomes

Although no statistically significant differences were observed between groups for vascular outcomes, there was a likely beneficial improvement for FMD (*p* = 0.203) and a possibly beneficial improvement in diastolic blood pressure (*p* = 0.293: Table 3) in the intervention group relative to controls. Intervention effects for systolic blood pressure and intima-media thickness were unclear or likely trivial, respectively.

#### Blood sampling

A beneficial reduction in total cholesterol was observed in the intervention group relative to the controls (*p* = 0.049: Table 3). Intervention effects for fasting plasma glucose or triglyceride concentrations were unclear.

#### Musculoskeletal and anthropometric outcomes

Although no statistically significant differences were observed between groups for musculoskeletal discomfort/pain, there was a likely beneficial reduction in the intervention group relative to controls for upper back discomfort/pain (*p* = 0.096) and a possibly beneficial reduction in neck and shoulder discomfort/pain (*p* = 0.155) (Table 3). No differences were observed between groups for body mass or BMI (results not shown).

#### Acceptability and feasibility

From the 19-item Likert scale, participants reported the sit-stand workstation easy to use and participants were comfortable using the workstation in front of others (Table 4). Most participants wanted further advice and guidance on how to use the workstation to optimise health. A majority of participants reported the sit-stand workstation did not decrease work-related productivity or interfere with completion of tasks. 33 % of participants reported more back pain on days when using the workstation and 66 % reported they would use the workstation at work if offered to them by their employer.

**Table 4:** Acceptability and feasibility of sit-stand workstations (1 = strongly disagree, 2 = disagree, 3 = neutral, 4 = agree, 5 = strongly agree)

Questions	Quartile 1	Median	Quartile 3
The sit-stand workstation is easy to use	4.0	4.0	5.0
I would use the sit-stand workstation as an alternative to be active on days that the weather is bad	2.0	3.0	4.0
I felt comfortable using the sit-stand workstation in the presence of others at my work	3.5	4.0	5.0
My work-related productivity decreased while using the sit-stand workstation	2.0	2.0	3.0
The quality of my work decreased while using the sit-stand workstation	2.0	2.0	3.0
The sit-stand workstation interfered with my daily work-related tasks	2.0	2.0	4.0
I could conduct normal computer-related tasks while using the sit-stand workstation	3.0	4.0	5.0
I could read comfortably while using the sit-stand workstation	2.3	4.0	4.0
I was more tired on days I used the sit-stand workstation	2.0	2.0	3.0
I had more back pain on days I used the sit-stand workstation	1.3	2.0	4.0
I had more joint pain on days I used the sit-stand workstation	2.0	2.0	2.0
I had more muscle aches on days I used the sit-stand workstation	1.3	2.0	3.0
My physical activity increased while at work as a result of the sit-stand workstation	3.0	4.0	4.8
The time I spent being sedentary decreased while at work as a result of the sit-stand workstation	3.0	4.0	5.0
My physical activity increased outside of work as a result of the sit-stand workstation	2.0	2.5	3.0
If I were offered a sit-stand workstation by my employer, I would use it while at work	2.3	4.0	5.0
I used the sit-stand workstation at consistent and regular intervals during the working day	2.0	3.0	4.0
I would welcome further advice and guidance for using the sit-stand workstation to optimise health gains	3.3	4.0	5.0
I would use a sit-stand workstation while at home	2.0	2.0	3.0

Participants approached and experienced sit-stand workstation use in a heterogeneous manner. Two broad themes emerged from interview data which were patterns of workstation use and factors that have the potential to influence workstation use. With reference to patterns of workstation use, findings demonstrated variation when participants were permitted to self-select the standing opportunities. Some participants reported having used the workstation for whole days:

*“I did start off the first week of using it [the sit-stand workstation] almost all of the day” (P7)*

*“The first day, 2 days, 3 days I used it [the sit-stand workstation] pretty much all the time” (P3).*

Whilst others reported self-selected patterning in relation to time of the day and hours/minutes of use:

*“I did try and use it [the sit-stand workstation] at first even like every half an hour or so, or like quite often and then if I sat down I would try and use it again in half an hour or so and then it got to, oh I’ll do 5 or 10 min every hour” (P4).*

Participant two described how they used the workstation a lot in the first instance, but use declined over time:

*“I tried sort of like to do at least an hour in the morning and an hour in the afternoon” (P2)*

*“I think towards the end I tend to sit down a lot … I think it [use of the sit-stand workstation] just tailed off in the end” (P2).*

Subthemes to the factors that influenced workstation use included workstation design (*n* = 3), work tasks (*n* = 5), the social environment (*n* = 5), habits (*n* = 5) and alertness (*n* = 4). Feedback suggested interplay between the workstation design and type of task that could be completed when in a standing position. For example, it was frequently reported that the workstation was too small and restrictive to completing tasks that required some desk space:

*“Depends what work you had on because we use files a lot and it [the sit-stand workstation] wasn’t you know very good for with files and things were you tend to sit down” (P2)*

*“Some duties couldn’t be performed you know with the device up you know maybe like filing you know different sort of paper based work” (P5)*

Further negative factors reported in relation to workstation design included the non-sturdy nature of the station:

*“I thought it [the sit-stand workstation] was a really poor design. Just the way it bounced about and the screen kept moving and cords getting in the way and all this” (P1)*

The social environment had both a positive and negative impact upon workstation use. Some participants noted trepidation and feeling self-conscious in an environment where colleagues were working in seated positions. In addition there was a degree of consideration of others in the environment as a result of them standing whilst others were sitting.

*“Initially we were like uh God I’m standing up everyone else is sitting down …….. some people just felt a bit self-conscious erm just because they were standing up and everyone else around them wasn’t maybe that made them feel uncomfortable (P3)*

*“If people were coming in to see these people (colleagues in close proximity) I sat down not to be a distraction so they can concentrate on what they’re doing” (P6)*

Findings also identified cases of support from peers using the desks and also prompting for such use in observing the behaviour of peers.

*“You would see someone else pop up and use theirs so you would think, oh yeah I’ll use mine” (P4)*

Once the initial novelty of using the standing workstation had worn off, participants identified difficulty in remembering to work in the standing position and reverted to old habits of sitting:

*“After a couple of weeks you started to decline, so there was almost like a novelty effect and then people were too busy to think about standing so they just reverted to sitting.” (P1)*

*“I got back into my old working habits of just sitting down again and then just sort of forgot that I had it and just I’d realise halfway through the afternoon that I hadn’t stood up today but I think like just back to old habits so you’re used to sitting down a lot.” (P2)*

Perceived concentration and alertness also potentially influenced workstation use, for example some participants indicated they felt better able to concentrate when sitting:

*“I don’t know whether standing up and being able to see everyone more was a bit of a distraction as well but yeah I did find I couldn’t concentrate as much [when standing] and I’d need I think it’s just a natural thing to sit down and have all your things around you” (P4).*

In contrast some participants observed heightened alertness and enhanced productivity when standing:

*“I did feel like a bit more awake” (P2)*

*“I think it [standing] kind of makes me more productive straight away” (P3).*

## Discussion

To our knowledge, this was the first RCT to use qualitative and quantitative methods to evaluate the feasibility and acceptability of sit-stand workstations and the impact on behavioural, cardiometabolic and musculoskeletal outcomes in asymptomatic office workers. The findings suggest that sit-stand workstations are a feasible tool for reducing daily sitting time and improving cardiometabolic risk over 8 weeks. Most participants self-reported that the workstation was easy to use and their work-related productivity did not decrease when using the device. Consistent with previous trials [11, 12, 22, 26] the magnitude of the decrease in sitting time was similar to the magnitude of the increase in standing time, and the variation in sitting reduction across studies (33–137 min/day) is likely due to different trial designs and the heterogeneity of samples and sitting time assessment method. Investigations on the long-term use of sit-stand workstations are now required to understand the sustainability of these changes in office behaviour.

Beneficial changes in cardiometabolic outcomes were found after only 8 weeks of using a sit-stand workstation. Observed reductions in total cholesterol are an important finding since total cholesterol concentration is positively associated with risk of developing coronary heart disease [47]. Similarly, the beneficial changes observed for FMD may have clinical importance, as FMD is a surrogate marker of vascular endothelial function that strongly and independently predicts future cardiovascular events [15, 16]. Finally, while the possibly beneficial change in diastolic blood pressure in the present trial is contrary to a trial that found a potentially negative change in diastolic blood pressure, that trial lasted only 4 weeks [12]. These collective findings for cholesterol, FMD and diastolic blood pressure imply that if the observed use of the workstation continued over a longer duration, sitting reduction via sit-stand workstations could have important ramifications for the prevention and reduction of cardiometabolic risk in a large proportion of the population [15, 16].

Findings from the musculoskeletal survey suggest sit-stand workstations did not increase discomfort or pain in the present sample. Instead, possibly beneficial reductions in upper back, and neck and shoulder discomfort/pain were observed. This is consistent with sit-stand workstation trials that observed reductions in upper back and neck pain [26] and decreased neck, knee and ankle/feet symptoms [22]. In contrast, one trial did report increased symptoms for shoulder pain after 12 weeks of sit-stand workstation use [22] and acceptability data in the present trial suggested some participants experienced discomfort when using the workstation, though this appears due to excessive and inappropriate use. Accordingly, while the majority of the literature suggests sit-stand workstations have little to no detrimental impact on musculoskeletal outcomes in the short term, a greater understanding of the long-term impact of sit-stand workstations on musculoskeletal outcomes is required, and providing workers with more comprehensive advice and guidance on safe and optimal use for health is supported by our findings.

The qualitative data provided new insights into participant’s experiences and barriers to sit-stand workstation use [20–22]. Patterns of workstation use varied across participants, and an interaction between workstation design and task type appeared to influence how participants made use of the standing feature. For example, completing paper-based and filing tasks were difficult when standing due to a lack of workstation space. Researchers and practitioners may wish to consider the work tasks typically completed by the targeted workforce when deciding the type of sit-stand workstation to implement in workplaces (e.g. sit-stand workstation vs. sit-stand desk). Future research could also examine changes in perceived concentration and alertness across task type over time, as some workers cited that they completed work in a seated posture to facilitate concentration and ultimately task completion.

The social environment and in particular the presence and actions of others were reported to support and deter standing work. For example, seeing co-workers standing prompted a transition from seated to standing work, while other workers cited feelings of self-consciousness as a reason to stay seated. Future trials could examine the effect of additional intervention strategies (e.g. workplace champions, wider workforce education on the intervention and its aims) to the simple provision of a standing workstation. Such strategies may additionally support sustained use of the workstation, and prevent workers from reverting back to the habit of seated working, which was observed in the present trial.

With regard to the design of future sit-stand workstation trials, sitting time data from this study has important implications. Previous parallel-group trials have used cluster randomisation to minimise treatment contamination on sitting and standing time between treatment arms. Though cluster randomisation is logical in multicomponent interventions that include organisational- and individual-level strategies, treatment contamination was previously unknown in single-level trials using sit-stand workstations. The current trial demonstrates that sitting time in the control group did not drop below baseline values over 8 weeks and statistically significant point estimates are comparable to a previous cluster RCT of similar design [11]. It is important to note that this study only measured one form of contamination, and did not for example assess if participants passed on ergonomic information or the workstation to control participants, however this is unlikely. Therefore, whilst further research is required to confirm these findings, the use of individual rather than cluster randomisation may be appropriate in future single-level trials that provide sit-stand workstations in an office setting. Individual compared to cluster randomisation will increase statistical power [23] and avoid factors associated with cluster randomisation, including recruitment bias, the need for larger samples, and the increased cost, length and complexity of a trial [48].

Strengths of this study were the RCT design, objective measurement of novel cardiometabolic outcomes, investigation of workstation acceptability and feasibility, control of confounding factors, and the use of magnitude-based inferences alongside traditional hypothesis testing. However, limitations were evident. Self-reporting activity places a higher burden on participants and may not produce estimates as accurate as accelerometers such as ActivPal. However, baseline sitting, standing and walking time were comparable to trials using accelerometers [11, 12, 22] and the current trial used more rigorous inclusion criteria for activity monitoring (≥2 valid days at each time point) than the aforementioned trials that had no minimum requirement for the number of valid days at any time point. The RCT, though of similar [11, 22] or greater [12, 26] duration to previous trials, did not investigate the long-term sustainability and impact of sit-stand workstations, or unintended changes in leisure time behaviour. Finally, a minority of participants were in health-related posts and a majority of participants were White British, non-smoking, normal weight women with a tertiary education qualification. While the sample may not be wholly representative of the population intended to be analysed, the sample held diverse office-based job roles across various departments, and baseline sitting time was comparable to other general-office based populations, [12, 49] increasing the generalizability of findings.

## Conclusion

Short-term use of a feasible sit-stand workstation reduced daily sitting time and led to beneficial improvements in cardiometabolic risk parameters in asymptomatic office workers. Most participants self-reported that the workstation was easy to use and their work-related productivity did not decrease when using the workstation, however factors reported to negatively influence workstation use included workstation design, the social environment, work tasks and habits. If the observed workstation use and health benefits are maintained or improved over longer periods, sit-stand workstations may offer health benefits for a large proportion of the working population and economic benefits for organisations through enhanced employee health and wellbeing. Research should elucidate whether long-term use of sit-stand workstations leads to sustained reductions in workplace sitting, further improvements in the reported surrogates of cardiovascular and metabolic disease risk, and economic benefits for organisations.

## Abbreviations

RCT: randomised controlled trial
HDL: high-density lipoprotein
EMA: ecological momentary assessment
FMD: flow-mediated dilation
cIMT: carotid artery intima media thickness
BMI: body mass index
SD: standard deviation
ANCOVA: analysis of covariance
CI: confidence interval
